# Supplementary material for: Helminth Interactions with Bacteria in the Host Gut Are Essential for Its Immunomodulatory Effect
Source: Microorganisms. 2021 Jan 22;9(2):226. doi: 10.3390/microorganisms9020226 (PMC7910914; doi:10.3390/microorganisms9020226)

**Supplementary data 2**

Clinical assessment of the rats` fitness over the course of experiment

| Day of experiment | % of rats with hematochezia | | | | Mean fecal consistency | | | |
| --- | --- | --- | --- | --- | --- | --- | --- | --- |
|  | HC | HAC | HA | AC | HC | HAC | HA | AC |
| 18 | 0 | 0 | 0 | 0 | 5 | 5 | 5 | 5 |
| 24 | 0 | 0 | 0 | 0 | 4.9 | 4.2 | 4.7 | 4.1 |
| 31 | 91 | 73 | 0 | 81 | 1.3 | 2.1 | 5 | 1.3 |

Weight change by the experimental groups over the course of experiment


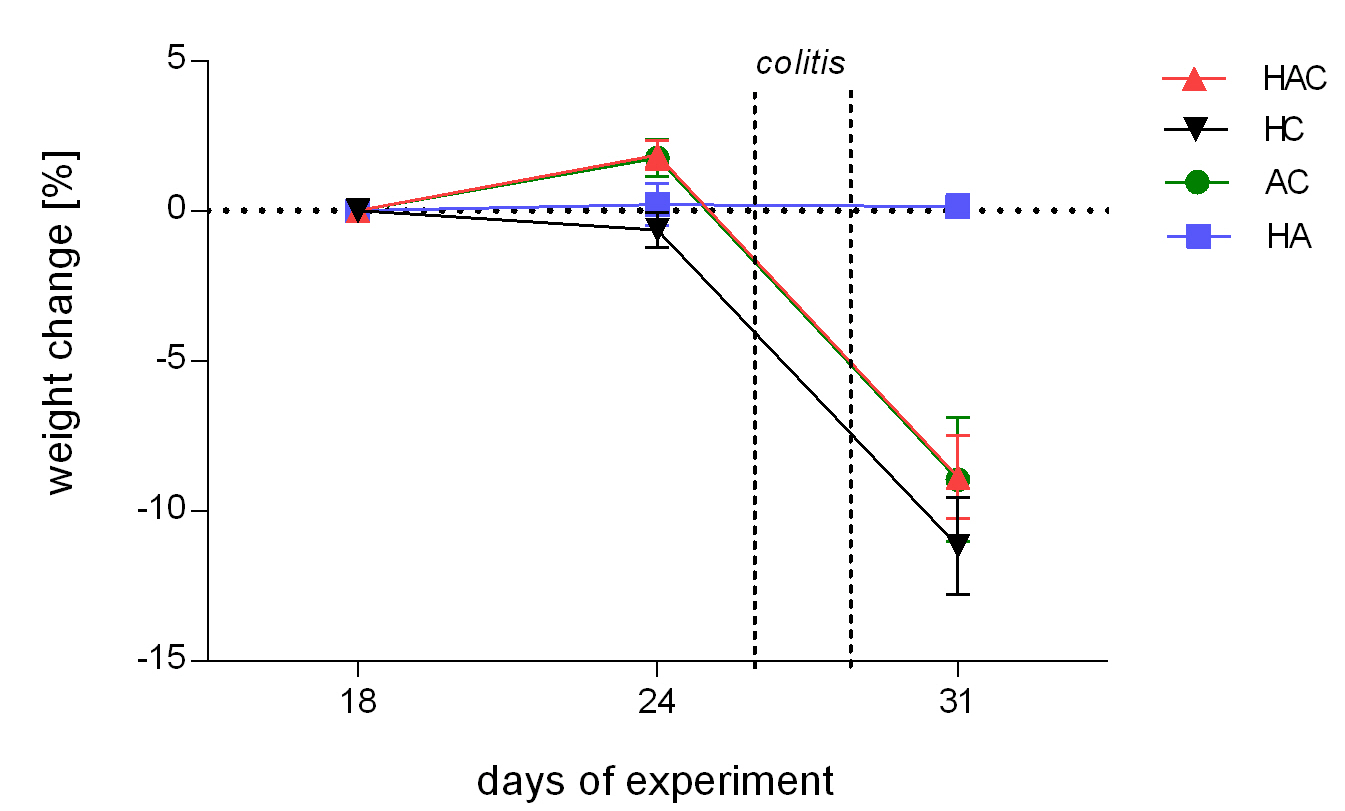

Supplement: Supplementary file 1 [file microorganisms-09-00226-s001.zip › SupplMaterial/Supplementary_data_2.docx]
