# Supplementary figures and images for: Helminth Interactions with Bacteria in the Host Gut Are Essential for Its Immunomodulatory Effect
Source: Microorganisms. 2021 Jan 22;9(2):226. doi: 10.3390/microorganisms9020226 (PMC7910914; doi:10.3390/microorganisms9020226)

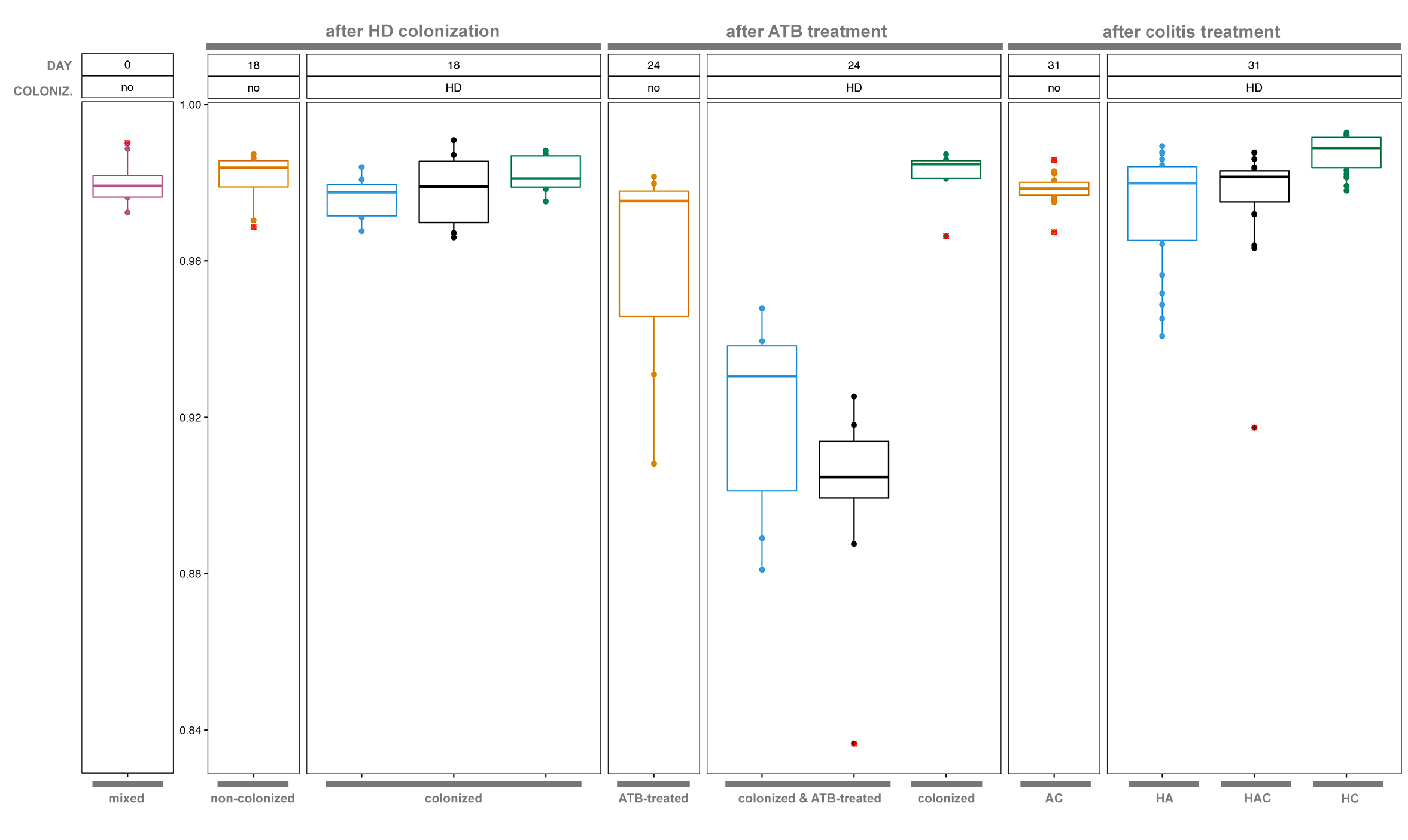

Supplement: Supplementary file 1 [file microorganisms-09-00226-s001.zip › SupplMaterial/Supplementary_data_1.jpg]
